# N-terminal truncation of STAT1 transcription factor causes CD3- and CD20-negative non-Hodgkin lymphoma through upregulation of STAT3-mediated oncogenic functions

**DOI:** 10.1186/s12964-025-02183-2

**Published:** 2025-04-26

**Authors:** Sana Mumtaz Sheikh, Julia Staab, Martina Bleyer, Aleksandar Ivetic, Fred Lühder, Oliver Wirths, Thomas Meyer

**Affiliations:** 1 Department of Psychosomatic Medicine and Psychotherapy, University Medical Center Göttingen, Göttingen, Germany; 2 Institute of Pathology, University Medical Center Düsseldorf, Düsseldorf, Germany; 3 German Primate Center, Leibniz Institute for Primate Research, Göttingen, Germany; 4 School of Cardiovascular and Metabolic Medicine & Sciences, BHF Centre of Research Excellence, King’s College London, London, United Kingdom; 5 Institute for Neuroimmunology and Multiple Sclerosis Research, University Medical Center Göttingen, Göttingen, Germany; 6 Department of Psychiatry and Psychotherapy, University Medical Center Göttingen, Göttingen, Germany

## Abstract

The cytokine-driven transcription factor STAT1 (signal transducer and activator of transcription 1) executes anti-microbial and pro-apoptotic functions, and loss-of-function mutations are associated with increased susceptibility to various infections and the development of tumors. A targeted mutation in mice expressing an N-terminally truncated STAT1 protein (STAT1-ΔN) typically develops splenomegaly in animals older than 6 months due to the formation of splenic non-Hodgkin lymphomas. The expression of the STAT1-ΔN variant resulted in the disruption of normal spleen architecture by malignant CD3- and CD20-negative tumor cells, which stained positively for both tyrosine-phosphorylated STAT1 and STAT3. Immunoblotting of lysates from isolated tumor cells revealed the cytokine-independent hyperphosphorylation of both STAT proteins, whereas the expression level of NF-κB was significantly reduced. Gel-shift assays showed that the DNA-binding activity of STAT1-ΔN was increased compared to the wild-type protein. This elevated level of tyrosine-phosphorylated STAT1-ΔN did not further increase upon stimulation of isolated tumor cells with either interferon-γ (IFNγ), lipopolysaccharide (LPS), or the combination of both. Since the truncation mutant was unable to accumulate in the nucleus upon cytokine stimulation, real-time PCR data from tumor tissue as well as from isolated, IFNγ/LPS-treated lymphoma cells demonstrated significantly reduced STAT1-regulated target gene expression despite its observed hyperphosphorylation. The nuclear import defect of tyrosine-phosphorylated STAT1-ΔN was associated with an elevated tyrosine-phosphorylation level of its antagonistic homolog STAT3, which is a known oncogene. These data demonstrate that the lack of STAT1 nuclear accumulation interferes with the functional balance between the two STAT proteins and, thereby, promotes the formation of phospho-STAT3-expressing CD3^-/-^ CD20^-/-^ non-Hodgkin lymphomas in the spleens of the diseased animals.

**Supplementary Information:**

The online version contains supplementary material available at 10.1186/s12964-025-02183-2.

## Introduction

The Janus kinase-signal transducer and activator of transcription (JAK-STAT) signaling pathway controls several essential biological functions in hematopoiesis, immune response, inflammation, tissue repair, apoptosis and proliferation [22]. Binding of extracellular cytokines, such as interferons (IFNs), interleukins (ILs), or colony-stimulating factors, to their cognate receptors results in tyrosine phosphorylation of the carboxy-terminal receptor chains by non-covalently bound JAK kinases. Subsequently, the activated JAKs phosphorylate STAT proteins at a single tyrosine residue in their carboxy terminus [22, 30]. The tyrosine-phosphorylated STAT molecules, which all share the same domain structure, are then actively transported against their concentration gradient into the nucleus, where they bind to cytokine-specific promoters and regulate gene expression [6].

Among the seven different STAT proteins identified in mammals, STAT1 and STAT3 are the most intensely studied family members given their prominent and partially antagonistic roles in immune reactions and cancer development. The founding member of this protein family, STAT1, regulates various cellular responses at both a transcriptional and non-transcriptional level and plays a central role in innate and adaptive immunity. Expression of STAT1 protects against invading microorganisms and triggers an immune response against transformed tumor cells [29]. In contrast to the tumor suppressor STAT1, which is a marker for favorable prognosis in different tumor types, the homologous STAT3 fulfills cell-autonomous functions in many cancer cells through maintaining stemcellness and promoting proliferation [13, 20, 35]. In several cancer types, the activity of the oncogenic STAT3 transcription factor is upregulated, which contributes to the malignancy of the disease [33].

Growing evidence suggests that STAT1 and STAT3 are indeed playing opposing roles in tumor development, cell proliferation, and cell survival. Mice expressing functionally inactive STAT1 display increased susceptibility to microbial infections [4, 22] and develop a variety of murine cancer entities [29, 30]. Porpaczy and colleagues demonstrated that an N-terminally truncated STAT1 mouse line (STAT1-∆N) resulted in spontaneous myeloid hyperplasia [34]. Moreover, transplantation of bone marrow from these animals induced lymphoma formation in donor mice, mimicking the aggressive B-cell lymphomas in patients with myelofibrosis treated with JAK1/2 inhibitors.

The mechanisms by which STAT1 inhibits tumor development may be related to both cytokine-dependent and -independent effects. Type I IFN-α is generally used for the treatment of skin cancers and various forms of leukemia, as it can lead to regression of the disease, probably due to apoptotic actions mediated by STAT1. The type II IFNγ is released at high concentrations locally at sites of a deregulated inflammation and functions as an essential element of tumor surveillance [8, 36, 39]. Spontaneously occurring or chemically induced tumors by methylcholanthrene are more often observed in mice lacking components of IFNγ signaling than in wild-type (WT) mice [18]. Mice deficient in IFNγ develop sarcomas or lymphomas more frequently than WT animals [22].

The experiments in this project were carried out using a mouse model with a deletion of the first three exons of the *Stat1*gene encoding an N-terminally truncated protein lacking 134 amino-acid residues, which leads to a functional knockout [3, 30]. In this article, we describe that this mutation results in impaired nuclear import of tyrosine-phosphorylated STAT1 and induces tumor formation in older mice. In addition, we examined the cross-talk between nuclear factor-κB (NF-κB) and STAT signaling pathways in the transformed cells.

## Materials and Methods

### Mouse model

The STAT1-∆N mouse line was purchased from Taconic Farms, Cologne, Germany, and crossbred over ten generations to a genetic C57BL6/N background (Charles Rivers, Sulzfeld, Germany). The mice were kept in individually ventilated cages in the animal house facility of the University Medical Center Göttingen. Kaplan-Meier survival curves for the two homozygous genotypes were generated and the estimates of the hazard functions were compared using the log-rank (Mantel-Cox) test. Animals died naturally or were euthanized when they developed signs of severe lymphoma. All experiments were conducted in accordance with a protocol approved by the authorities responsible for animal experimentation. The mice used for the experiments were between 6 and 8 months old and of both sexes.

### Immunohistochemical staining

Immunohistochemical staining was performed using tissue samples from liver, spleen, and bone marrow obtained from mutant mice. Specimens were fixed in 4% neutral buffered formalin and embedded in paraffin. Histological sections with a thickness of 3 to 5 μm were sliced from paraffin-embedded tissue blocks and deparaffinised using xylene, followed by dehydration in a series of graded alcohol. The rehydrated sections were then heat-treated in citrate buffer (10 mM citrate, pH 6.0) for 15 min and incubated with peroxidase blocking solution (3% H_2_O_2_ in phosphate-buffered saline [PBS]) at 4 °C for 15 min. The following monoclonal primary antibodies diluted 1:250 were used: phospho-STAT1-Tyr701 (58D6), STAT1 (D1K9Y), phospho-STAT3-Tyr705 (D3A7) (all from Cell Signaling Technology, Danvers, USA), phospho-STAT1-Ser727 (sc-16570), and phospho-STAT3-Ser727 (sc-136193) (both from Santa Cruz, Dallas, USA), whereas the antibody against STAT3 (D1B2J) was diluted 1:500. Antibodies directed against nuclear factor κB (NF-κB) p65 (D14E12), inhibitor of κBα (IκBα) (44D4), and inducible nitric oxide synthase (iNOS) (sc-8310) were purchased from Cell Signaling Technology and diluted 1:200 in 4% bovine serum albumin/phosphate-buffered saline (BSA/PBS). For the staining of CD3 (sc-20047 and sc-101442) and CD20 (Agilent Dako, M0755, clone L26, Santa Clara, USA), antibodies were diluted 1:200 in 4% BSA/PBS solution. Bound immunoreactivity was detected using the ABC method with biotinylated secondary antibodies followed by avidin-horseradish peroxidase complexes. Diaminobenzidine was used as a substrate to visualize the enzyme reaction, resulting in a brown reaction product. Finally, the serial sections were counterstained with Mayer’s hematoxylin, dehydrated, and mounted. Images were obtained using a light microscope (Zeiss, Oberkochen, Germany). The intensity of staining for each target antigen was assessed using a four-level semiquantitative grading system ranging from 1 (virtually no immunostaining) to 4 (strongest expression level). Histopathological evaluation was performed by two investigators who were blinded to the clinical data of the cohort.

### Isolation and treatment of spleen cells

Suspension cells were isolated from splenic tumors of mutant mice and from normal spleen tissue of their WT littermates. To this end, animals were euthanized by CO_2_ inhalation and their spleens were harvested and stored for preservation in ice-cold Dulbecco’s modified Eagle medium (DMEM) with 10% fetal calf serum (FCS) supplemented with glutamine, penicillin/streptomycin (100 IU/ml) and HEPES (25 mM). The spleen was subsequently smashed through a cell strainer to separate the cells and remove the connective tissue. The isolated cells were centrifuged at 1200 rpm for 6 min at 4 °C, and the pellet was rinsed once in FCS-supplemented DMEM. The suspended cells were then counted using a Neubauer chamber and plated in 6-well dishes for further analysis. Finally, the cells were either left untreated or treated with 50 ng/ml of murine IFNγ (Biomol, Hamburg, Germany) and/or 10 µg/ml of lipopolysaccharide (LPS, Sigma-Aldrich, Darmstadt, Germany) for 30 min, before cellular proteins were extracted.

### Protein extraction and Western blotting

Cells were harvested from stimulated splenic cell suspensions by centrifugation (2 min, 16000 g, 4 °C). The supernatant was discarded, and the pellet was rinsed once with PBS. The pellet of the second centrifugation step was lysed in 60 μl cytosolic extraction buffer (10 mM KCI, 20 mM HEPES, pH 7.4, 10% [v/v] glycerol, 0.1 mM Na_3_VO_4_, 1 mM EDTA, 0.1% IGEPAL-CA360, 0.4 mM Pefabloc [Sigma-Aldrich], 3 mM 1,4-dithiothreitol [DTT], and Complete Mini protease inhibitors [Roche]) and incubated for 5 min on ice, followed by a centrifugation step at 16000 g for 30 s at 4 °C. The supernatants were centrifuged again for 5 min at 16000 g and 4 °C, and the resulting supernatants were transferred to new 1.5 ml reaction tubes as cytoplasmic extracts. The pellets obtained after the third centrifugation step were resuspended in 60 μl of nuclear extraction buffer (420 mM KCl, 20 mM HEPES, pH 7.4, 20% [v/v] glycerol, 0.1 mM Na_3_VO_4_, 1 mM EDTA, 0.4 mM Pefabloc, 3 mM DTT, and Complete Mini protease inhibitors). After incubating for 30 min on ice, the extracts were centrifuged at 16000 g for 15 min at 4 °C. The resulting supernatants were then combined with the same volume of the corresponding cytoplasmic extracts to obtain whole cell lysates. For electrophoretic mobility shift assay (EMSA), protein extracts were stored at − 80°C without further processing. For Western blot analysis, whole protein extracts were heated for 3 min in 6x Laemmli buffer (350 mM Tris-HCl, 8% SDS, 30% glycerol, 10% mercaptoethanol, 0.04% bromophenol, pH 7.4) and proteins were separated on 10% SDS-PAGE (sodium dodecyl sulphate-polyacrylamide gel electrophoresis) gels. Proteins were then transferred onto a polyvinylidene fluoride (PVDF) membrane in a semi-dry blotting apparatus. After blocking with 4% BSA in TBS-T (Tris-buffered saline supplemented with 0.1% Tween-20), the membrane was incubated with monoclonal antibodies. To test for equal protein loading, the membranes were re-exposed with the GAPDH monoclonal antibody 14C10 (Cell Signaling Technology). Finally, the membranes were exposed to a secondary antibody conjugated with IRDye 800 CW (LI-COR, Bad Homburg, Germany), followed by four washing steps with TBS-T and a final one with TBS. Bound immunoreactivity was detected using the LI-COR Odyssey Imaging System.

### Electrophoretic mobility shift assay

For EMSA, two oligonucleotides with reverse complementary sequences and poly-T overhangs at their 5’ position were hybridized in a water bath at 95 °C. The M67 duplex oligonucleotide contains a single gamma-activated site (GAS) for STAT binding of the sequence 5′-TTTTCGACATTTCCCGTAAATCTG-′3. Radioactive labeling was carried out by a filling reaction using the Klenow fragment. For this purpose, 5 units of Klenow fragment derived from *E. coil* DNA polymerase I were mixed with 5 μL of 10x Eco-Pol buffer and combined with 0.1 ng of double-stranded oligonucleotides and 8 μL of [^33^P]-labeled adenosine triphosphate (ATP). The reaction mixture was then incubated at room temperature for 25 min. The reaction was stopped immediately by adding 1 μl of 0.5 mM EDTA solution and subsequently subjected to size-exclusion chromatography, including a final centrifugation step at 700 g for 3 min to reduce the concentration of free nucleotides.

### Real-time PCR

RNA was extracted from cultured cells and tissues obtained from WT and mutant mice. Isolated tumor and normal cells were either left untreated or treated with 50 ng/ml of IFNγ and/or 10 µg/ml of LPS for 6 h. PeqGold Total RNA kit (VWR Life Sciences, Darmstadt, Germany) was used to extract RNA followed by cDNA synthesis using the Verso cDNA Synthesis kit (Thermo Fisher Scientific, Waltham, MA, USA). Real-time PCR reactions were carried out in a volume of 20 μl, containing 25 ng cDNA, 10 μl of Absolute Blue qPCR SYBR Green Mix (Thermo Fisher Scientific), and 70 nM of each specific primer. The primer pairs used are listed in the Supplemental Table 1. The protocol used included a denaturation step at 95 °C for 15 min, followed by 40 cycles of denaturation at 95 °C for 15 s, annealing at 55 ^o^C for 30 s, and elongation at 72 ^o^C for 30 s. After the final elongation step, melting curve analysis was performed. Each reaction was analyzed in at least two different experiments in duplicates. *Gapdh* was used as a housekeeping gene to normalize the relative expression of STAT1-regulated target genes for each sample based on the ∆∆C_T_ method. The fold induction of STAT1-target genes normalized to *Gapdh* expression was calculated.

### Library preparation and RNA-seq

RNA was isolated from cultured cells of WT (*n*= 3) and mutant mice (*n*= 3) using TRIzol reagent (Thermo Fisher Scientific). Samples were stored at − 80°C for further analysis. RNA sequencing was performed at the Microarray and Deep-Sequencing Facility in the Department of Transcriptome and Genome Analysis Laboratory (TAL), Göttingen, Germany. The amount and quality of total RNA were measured using a Fragment Analyzer (Advanced Analytical Technology, Santa Clara, CA, USA) and the Standard Sensitivity RNA Analysis Kit (DNF-471). The RNA-seq library was prepared from 500 ng of total RNA (> 8 RNA integrity number) using an Illumina TruSeq stranded mRNA preparation kit. An improved strand-specific, massively parallel cDNA sequencing protocol was used. The QuantiFluor dsDNA system (Promega, Madison, WI, USA) was used to quantify libraries that were further pooled and sequenced using an Illumina HiSeq 4000 generating 50 bp single end reads (30–40 million reads/sample). Reads were aligned to the mouse reference genome (GRCm38, mus_musculus_mm10) for each sample using Staraligner software, with a maximum of allowing two mismatches per 50-bp read. The number of reads aligning to each gene was determined using featureCounts (version 1.4.5-p1) [24]. Differential expression analysis was performed using the Benjamini-Hochberg method implemented in the R/Bioconductor package DESeq2 (version 3.4.2) [25]. The Gene Ontology (GO) terms and Kyoto Encyclopedia of Genes and Genomes (KEGG) pathways were identified using WebGestalt software, and heat maps were generated using the web-based application for differential expression and pathway analysis of RNA-seq termed iDEP.951 [17].

### Fluorescence microscopy

Immunocytochemical experiments were performed using mesenchymal embryonic fibroblasts (MEFs) expressing either STAT1-WT or -∆N. Cells from both genotypes seeded on 8-well chamber slides were first treated for 30 min with LPS, IFNγ, or LPS/IFNγ, and then fixed with methanol for 20 min at − 20 °C. After washing twice in PBS, cells were permeabilized using 1.0% Triton X-100/PBS and subsequently incubated in 25% FCS/PBS to block non-specific binding sites for 45 min at room temperature. Cells were stained with the pan-STAT1 antibody D1K9Y diluted 1:1000 in 25% FCS/PBS for 45 min. After washing three times in PBS, a Cy3-conjugated secondary antibody (Dianova, Hamburg, Germany) diluted 1:500 in 25% FCS/PBS was used to incubate the cells for 45 min, followed by Hoechst 33342 staining (5 min). Finally, the samples were mounted and images were captured using a Nikon Eclipse Ti fluorescence microscope (Nikon Instruments, Amsterdam, Netherlands). ImageJ software from the National Institutes of Health (NIH) was used to measure the fluorescence intensities in both the cytoplasm and nucleus.

### Data analysis

Statistical data were analyzed using GraphPad PRISM and are expressed as means ± standard deviations. Differences between groups were assessed using Student’s *t*-test. All experiments were performed in triplicate. A p value of ≤ 0.05 was chosen as the significance level. Figures were designed using CorelDRAW Graphics Suite 2023.

## Results

### STAT1-∆N mice demonstrate splenomegaly due to tumor formation

**Fig. 1:**
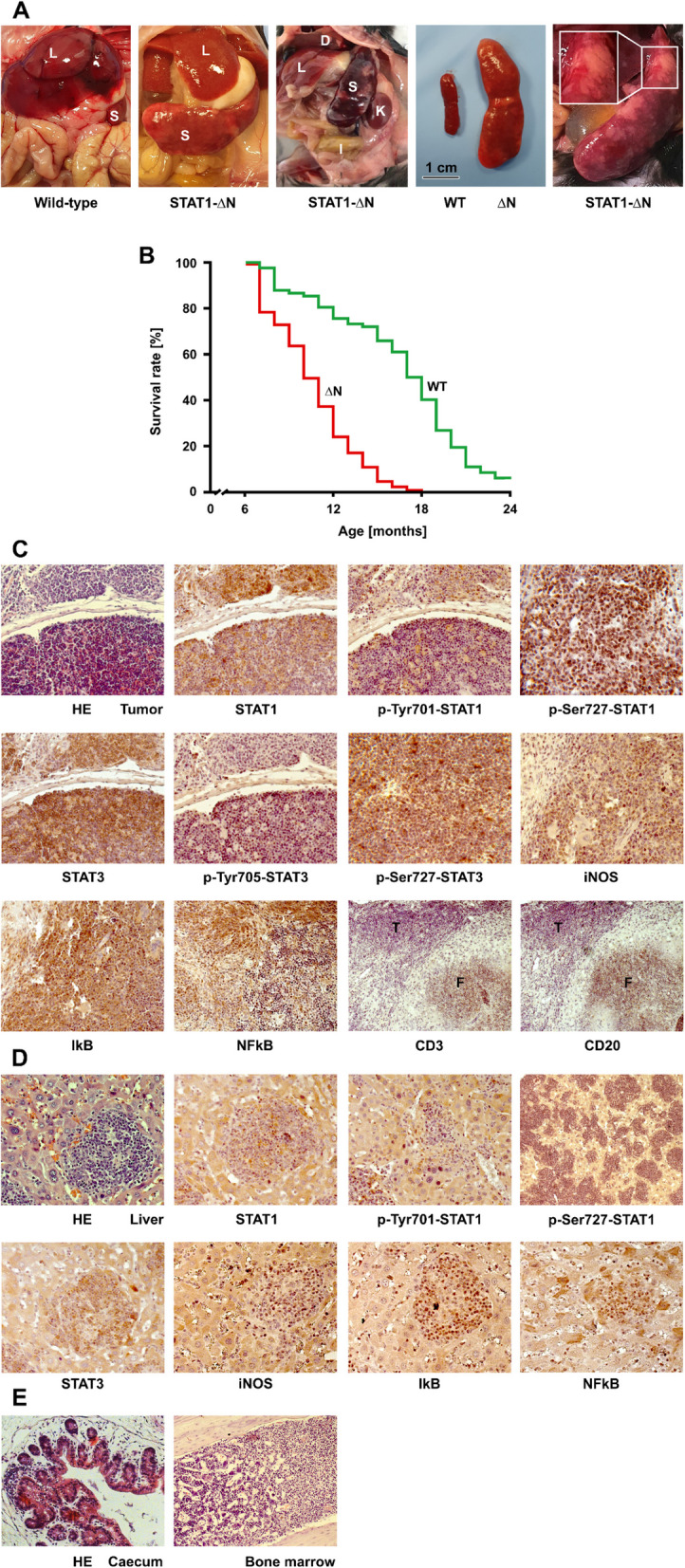
Pathological assessment of splenic tumor tissue from older STAT1-∆N-expressing mice. **A** *In situ* localization of the enlarged spleen (S) and liver (L) in STAT1-∆N-expressing mice compared to their wild-type (WT) littermates (*n*= 5 each). STAT1-∆N mice showed excessive splenomegaly with a much larger and heavier spleen (> 2 g) than in WT mice. Laparotomy of a STAT1-∆N mouse demonstrated the enlarged spleen (S) in relation to other abdominal or retroperitoneal organs such as liver (L), left kidney (K), diaphragm (D) and intestine (I). **B** Survival analysis using Kaplan-Meier curves was conducted to compare mice lacking the N-terminal domain of STAT1 (*n*= 129) with their WT counterparts (*n*= 82). The results showed a significantly reduced survival among the mice expressing the N-terminally truncated STAT1 (*p*< 0.001). **C** Histopathological spleen sections from transgenic STAT1-∆N-expressing mice were stained either conventionally with hematoxylin and eosin (HE) or immunohistochemically with antibodies directed against CD3, CD20, p-STAT1, p-Ser727-STAT1, STAT1, p-STAT3, p-Ser727-STAT3, STAT3, iNOS, NF-κB, and IκBα, as indicated. The CD3- and CD20-stained tissue sections showed no specific labeling in tumor cells (T) but positive immunoreactivity in cells from normal follicles (F). **D** Representative images of liver sections from STAT1-∆N-expressing mice stained with the indicated antibodies. **E** Histologically, there were signs of mild to moderate acute hemorrhagic and necrotizing inflammation with submucosal edema and intraluminal bacteria in the cecum of a STAT1-∆N-expressing animal (left image). Samples from bone marrow showed increased cellularity with a shift towards myeloid cells, indicating a leukemic reaction (right image)

At an age of 6 months or older, STAT1-∆N-expressing mice spontaneously developed excessive splenomegaly (> 2 g) due to the formation of multifocal extranodal tumors within the enlarged spleen (Fig. 1A). The normal architecture of the spleen was disrupted by invading tumor cells, but there were no obvious signs of extramedullary hematopoiesis. In contrast, mice expressing the WT protein showed no tumor formation in the spleen (Fig. 1A). Kaplan-Meier survival analysis of 129 STAT1-∆N-expressing and 82 STAT1-WT-expressing mice showed that animals lacking the N-terminal domain of STAT1 had a significantly shorter life expectancy than their WT littermates (*p*< 0.001) (Fig. 1B). With the exception of hepatosplenomegaly, no alterations were observed in the size and weight of the kidneys, lung, heart, and brain between the transgenic mice and their WT littermates.

Histopathological data obtained from mutant mice showed a follicular pattern and nodular appearance in spleen sections [12], with interspersed multinodular growth of medium-sized tumor cells. Hematoxylin-eosin staining of the enlarged STAT1-∆N-expressing spleens demonstrated a markedly irregular pattern of tumor cells, exhibiting a thin rim of cytoplasm with small round nucleoli (Fig. 1C). The CD3- and CD20-negative splenic tumor cells exhibited immunopositivity for both tyrosine-phosphorylated STAT1 and tyrosine-phosphorylated STAT3 (Fig. 1C). In addition, tumor cells displayed serine phosphorylation at position 727 in both STAT1 and STAT3, as determined by immunostaining with phospho-serine-specific antibodies directed against STAT1 and STAT3, respectively (Fig. 1C). However, the expression of NF-κB in extranodal tumor foci was significantly reduced compared to that in areas with no tumor cell formation (Fig. 1C). In liver metastasis, we observed a reduction in tyrosine phosphorylation of STAT1 and slightly elevated STAT3 levels (Fig. 1D). Compared to adjacent, non-transformed hepatocytes, tumor cells showed increased NF-κB and IκBα expression and slightly reduced iNOS staining intensity. Splenic tumors clinically resemble the histopathological features of non-Hodgkin lymphoma (NHL).

As demonstrated in Fig. 1E, tissue specimens obtained from the cecum of a STAT1-∆N-expressing mouse showed multifocal acute hemorrhagic necrotizing typhlitis with submucosal edema and probably dysbacteriosis. Segmental, high-grade acute hemorrhagic necrosis characterized by localized tissue destruction was observed, indicating disintegration of the epithelial barrier. Dysbacteriosis in the lumen of the colon suggested an imbalance in the composition of the intestinal flora, accompanied by the presence of infiltrating immune cells in the mucosa. Gram-positive bacteria were found not only in the crypts but also in areas of necrotic tissue within the intestinal wall. These submucosal inflammatory infiltrates were characterized by an accumulation of neutrophil granulocytes. Neutrophil leukocytostasis and prominent submucosal edema resulted in marked enlargement of layers beneath the mucosa. An examination of the bone marrow showed increased cellularity with a shift towards myeloid cells, indicating a leukemic reaction (Fig. 1E). In summary, these findings support a diagnosis of severe multifocal acute hemorrhagic necrotizing typhlitis with submucosal edema and dysbacteriosis.

### Hyperphosphorylation of STAT1-∆N and STAT3 in tumor cells

**Fig. 2:**
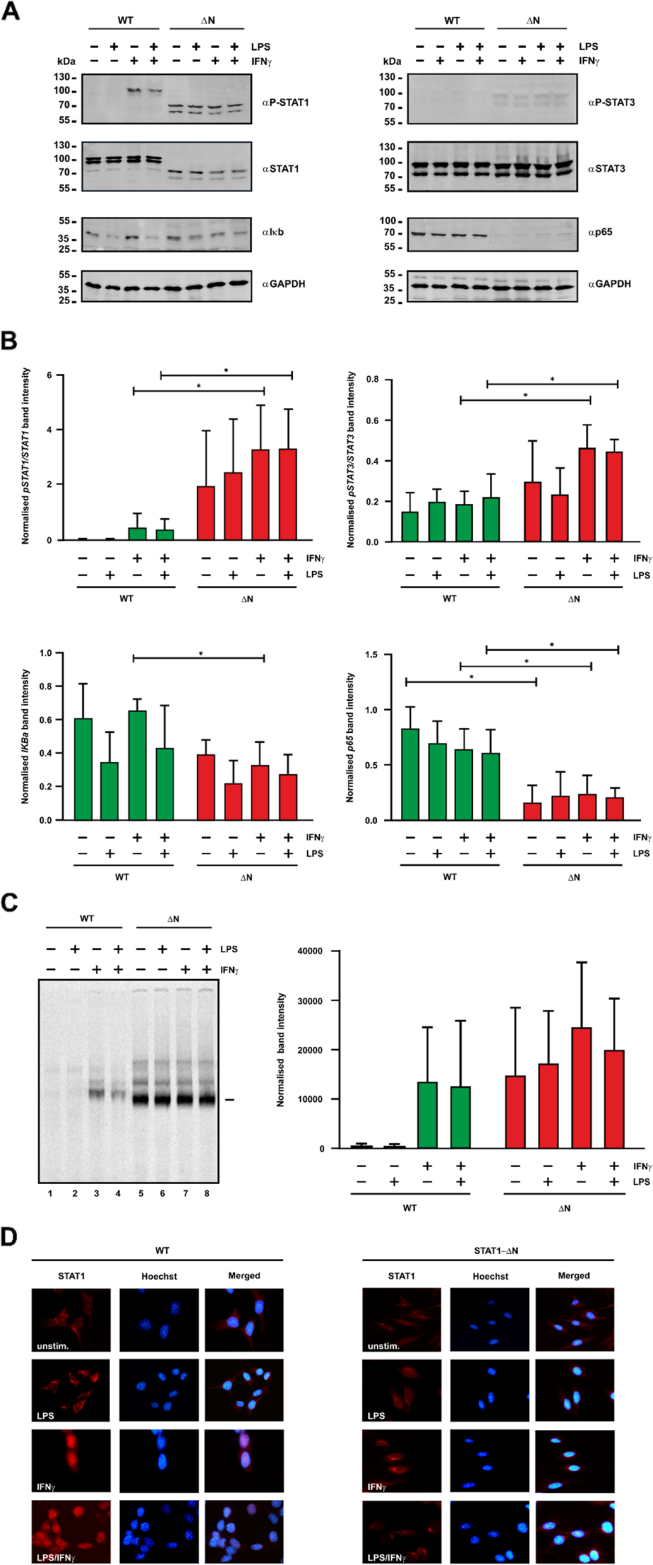
Increased tyrosine phosphorylation and impaired nuclear accumulation of STAT1-∆N. **A** Isolated splenocytes from WT mice and tumor cells from STAT1-∆N mice were treated with 50 ng/ml of murine IFNγ and 10 mg/ml of LPS for 30 min. Representative Western blot data from whole protein extracts were stained with p-STAT1, STAT1, p-STAT3, STAT3, NF-κB, and IκBα antibodies, as indicated. **B** Western blot bands were quantified using ImageJ software from three independent experiments. Asterisks indicate significant differences between the two genotypes depending on the treatment condition. **C** EMSA with total protein extracts from splenocytes unstimulated or stimulated with LPS and/or IFNγ were incubated with a radioactively [^33^P]-labeled M67 probe that contains a single consensus GAS motif. The autoradiogram (left) shows a representative result from one of the three independent gel-shift assays used for quantification (right). **D** Reduced nuclear accumulation of tyrosine-phosphorylated STAT1-∆N following cytokine stimulation. Immunocytochemical experiments were performed using mesenchymal embryonic fibroblasts expressing STAT1-WT or STAT1-∆N; the samples were additionally stained with Hoechst dye for nuclear localization. Fluorescence micrographs show the intracellular distribution of pan-STAT1 in cells expressing the WT protein or its N-terminally truncated variant

To determine the degree of tyrosine phosphorylation of STAT1 and STAT3 in cultured tumor cells compared to normal controls, we performed Western blot experiments. To this end, cells were isolated from splenic tumors of STAT1-∆N-expressing mice and normal spleens of WT mice and subsequently treated for 30 min with 50 ng/ml of murine IFNγ and/or 10 µg/ml of LPS before protein extraction. The isolated tumor cells showed a constitutive hyperphosphorylation of the truncated STAT1 mutant, as well as STAT3, even before cytokine stimulation (Fig. 2A, B). Notably, the levels of tyrosine-phosphorylated STAT1 did not further increase when cells were stimulated with IFNγ, LPS, or a combination of both, as shown by Western blotting in Fig. 2A, B. NF-κB (p65) expression was reduced in tumor cells compared to that in cells from WT animals. There was a degradation of IκBα in both WT and STAT1-∆N-expressing mice. Quantification of data confirmed elevated levels of tyrosine phosphorylation of both STAT1 and STAT3 and reduced levels of both NF-κB and IκBα in lymphoma cells from mice expressing STAT1-∆N (Fig. 2B).

### Enhanced DNA-binding activity of STAT1-∆N mice in tumor cells

Furthermore, we examined the DNA-binding activity of mutant and WT STAT1 using EMSA experiments with whole protein extracts from NHL cells and normal splenocytes as controls. We observed that both the WT protein and the STAT1-∆N mutant bound to a single GAS site and, in addition, found enhanced DNA-binding activity of the mutant as compared to the WT protein in non-transformed cells (Fig. 2C). Notably, the STAT1 mutant showed detectable binding to the radioactively labeled GAS site even before cytokine stimulation. These results were consistent with the immunoblotting results (Fig. 2A, B), indicating elevated STAT1 activation in tumor cells already before *in vitro* cytokine exposure (Fig. 2C).

### STAT1-∆N shows a lack of nuclear accumulation upon stimulation of cells with LPS/IFNγ

Immunocytochemical staining of MEFs expressing either STAT1-WT or -∆N were performed to assess changes in the intracellular localization of STAT1 upon treatment with LPS, IFNγ and LPS/IFNγ, respectively (Fig. 2D). Immunofluorescence data revealed that treatment of STAT1-WT- and -∆N-expressing cells with LPS did not lead to accumulation of STAT1 in the nucleus. In contrast to the nuclear localization of STAT1 in WT-expressing MEFs, mutant STAT1 was unable to enter the nucleus upon cytokine stimulation.

### Reduced STAT1-regulated gene expression in isolated tumor cells

**Fig. 3:**
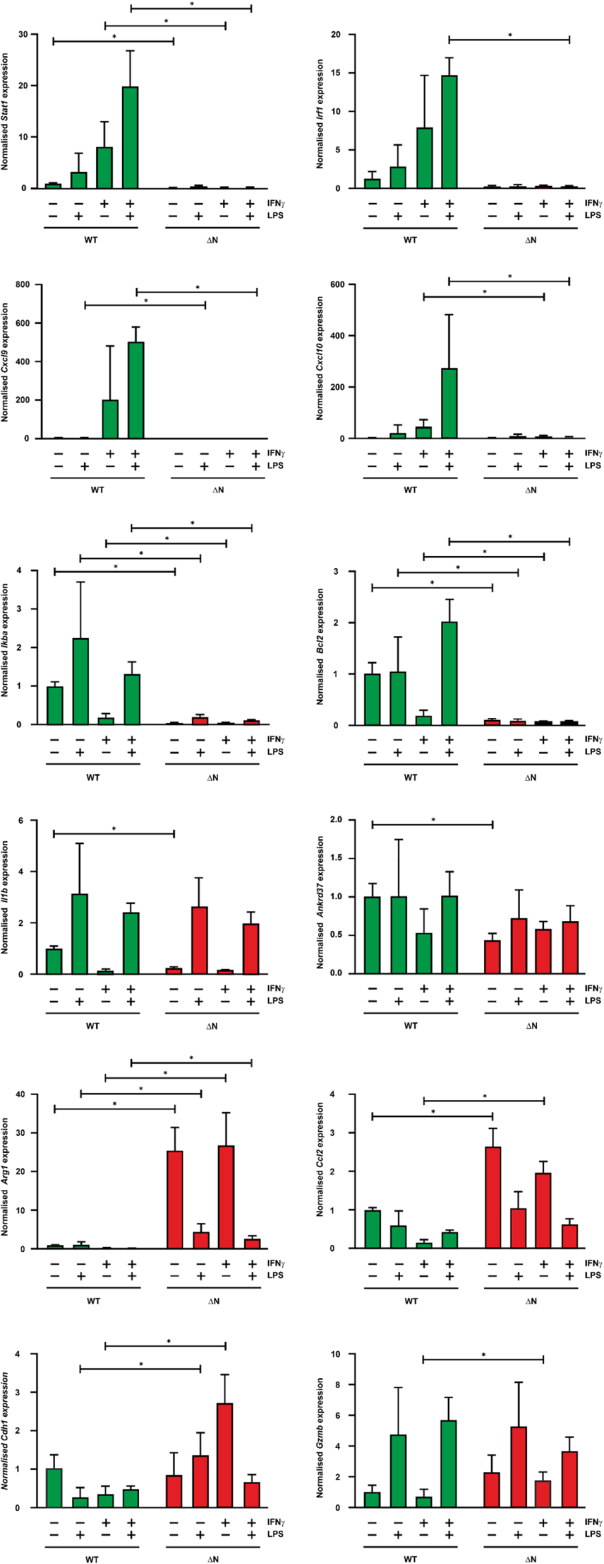
Reduced STAT1 target gene expression in lymphoma cells. Expression of STAT1-regulated target genes was determined by quantitative real-time PCR using isolated cells from normal spleen tissue of WT-expressing animals versus lymphoma tissue of STAT1-∆N-expressing mice. Plots show mRNA expression levels of *Stat1, Irf1, Cxcl9, Cxcl10, Iκbα, Bcl2, IL1b, Ankrd37, Arg1, Ccl2, Cdh1,* and *Gzmb* genes before and after 6 h of stimulation with LPS, IFNγ, and LPS/IFNγ. All genes were normalized to the expression of the house-keeping gene *Gapdh*. The bar graphs show standard deviations and mean values; significant differences between the WT protein and STAT1-∆N mutant are indicated by asterisks. Experiments were performed in triplicates. Data were analyzed using a two-tailed Student’s *t*-test with a *p*-value of *p< 0.05

Given the enhanced activation pattern of STAT1-∆N with respect to tyrosine phosphorylation, we subsequently investigated the mRNA expression of STAT-mediated target genes in tumor cells isolated from transgenic mice compared to cultured non-transformed cells from WT animals. To this end, cultured cells were stimulated *in vitro* with IFNγ, LPS, or a combination of both for 6 h (Fig. 3). Using real-time PCR, we analyzed a set of 12 STAT-regulated target genes at the transcriptional level in both NHL cells and normal controls*.* Results showed that upon cytokine stimulation most of the studied genes were expressed at a lower level in the tumor cells than in control cells, such as *Stat1*, *Irf1, Cxcl9*, *Cxcl10*, *IκBα*, and *Bcl2* (*p*< 0.05). However, in NHL cells, three genes were expressed at a higher rate than in control cells, namely *Arg1*, *Ccl2*, and *Cdh1*, which are all regarded being mainly under the transcriptional regulation of STAT3 [21]. In contrast to the above-mentioned STAT1-regulated target genes, these three STAT3-dependent genes were upregulated upon cytokine stimulation. We concluded that, in the comparison between cytokine-stimulated NHL cells expressing mutant STAT1 and WT-expressing control cells, there was a decrease in STAT1-mediated gene expression in the tumor cells, whereas genes upregulated by STAT3 were induced at higher rates.

### Gene set enrichment analysis shows differentially activated pathways in NHL

**Fig. 4:**
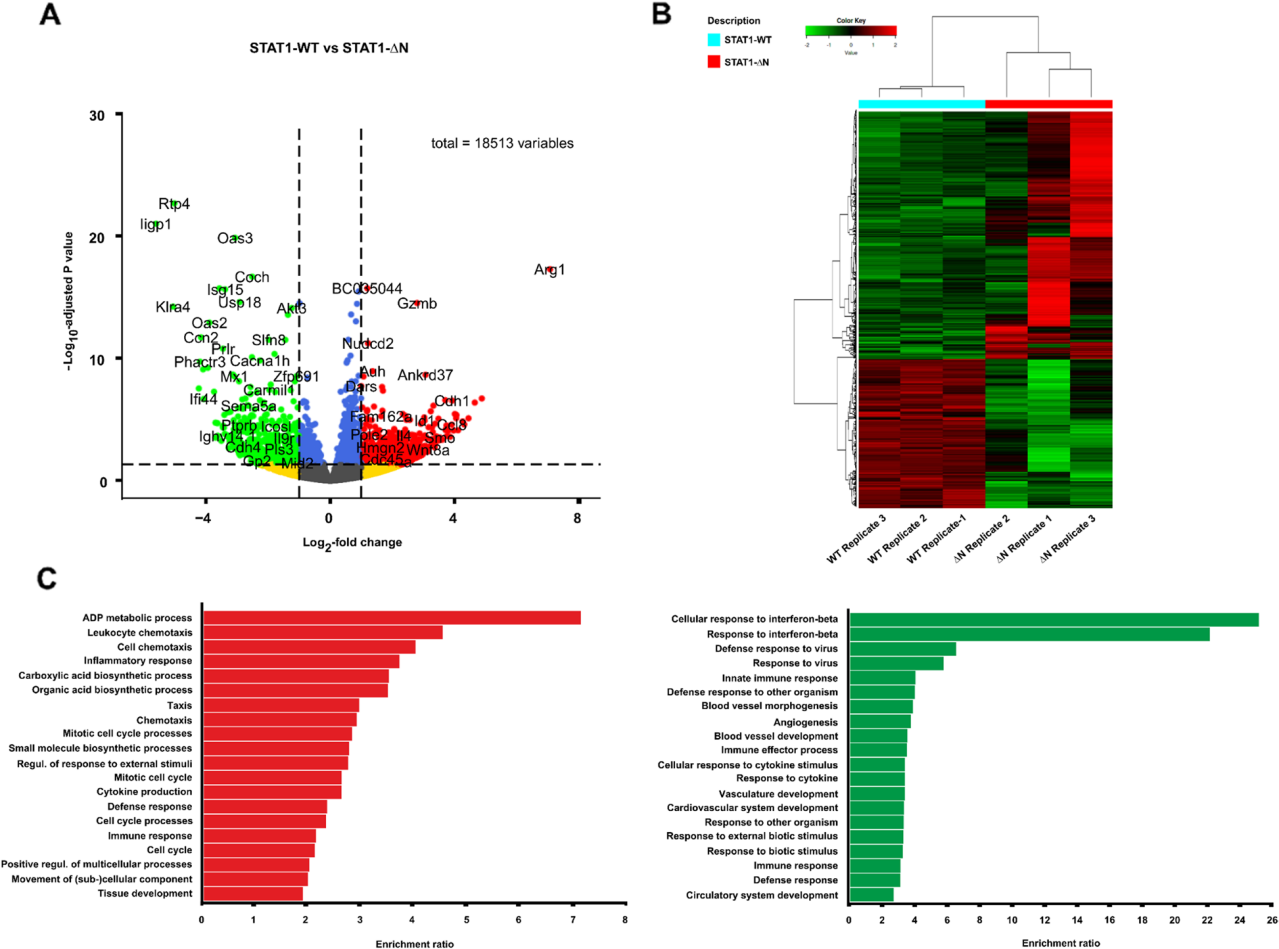
Gene set enrichment analysis shows unique pathways in non-Hodgkin lymphoma. **A** Volcano plot representation of DEGs in NHL compared to normal tissue.** B** Hierarchical clustering of differentially expressed genes (DEGs) in non-Hodgkin lymphomas (NHL) from STAT1-∆N-expressing mice versus normal spleen tissue from WT littermates. **C** List of the top 20 Gene Ontology (GO) terms related to biological processes (BP) for differentially expressed genes between normal spleen tissue and lymphoma.

Finally, we aimed to investigate differentially activated signal transduction pathways in NHL cells using RNA-seq technology. To this end, cDNA was synthesized from mRNA using freshly cultured tumor cells from transgenic mice expressing STAT1-∆N versus normal splenocytes from WT animals. Using a strand-specific, massively parallel sequencing technique for cDNA libraries, a total number of 18,513 genes were identified (Fig. 4A). Of these, there were 1498 differentially expressed genes (DEGs), including 924 that were upregulated and 574 that were downregulated in the comparison between the two genotypes. The corresponding volcano plot for the comparison between the two genotypes is shown in Figure 4A. The STAT3 target gene *Arg1* was the most upregulated gene with a fold change of more than 100, followed by *Cdh1* and *Ccl8*. Hierarchical clustering was performed based on the values of candidate genes for the two genotypes. We observed a higher number of upregulated genes in NHL cells than in normal cells (Fig. 4B).

To identify the biological processes in which these DEGs are involved, we performed GO enrichment analysis. The DEGs were classified into numerous biological processes, including leukocyte chemotaxis, inflammatory and defense processes, as well as immune responses (Fig. 4C). Kyoto Encyclopedia of Genes and Genomes (KEGG) analysis revealed the involvement of various immune response, cancer, and signal transduction pathways, including cytokines and cytokine-receptor interactions, which differed between STAT1-∆N-expressing NHL cells and normal WT cells (Supplemental Fig. 1A, B). Most of the genes involved in cytokine-cytokine receptor interactions were upregulated in NHL cells (colored red in Supplemental Fig. 1B). As expected, we found that components of metabolic and cancer pathways were among the most enriched pathways involved in NHL cells. In summary, the transcriptomic data show that NHL cells have a unique activation profile which differs from non-transformed cells in terms of the gene products that trigger cytokine responses.

### Reduced STAT1-regulated gene expression in NHL tissue

**Fig. 5:**
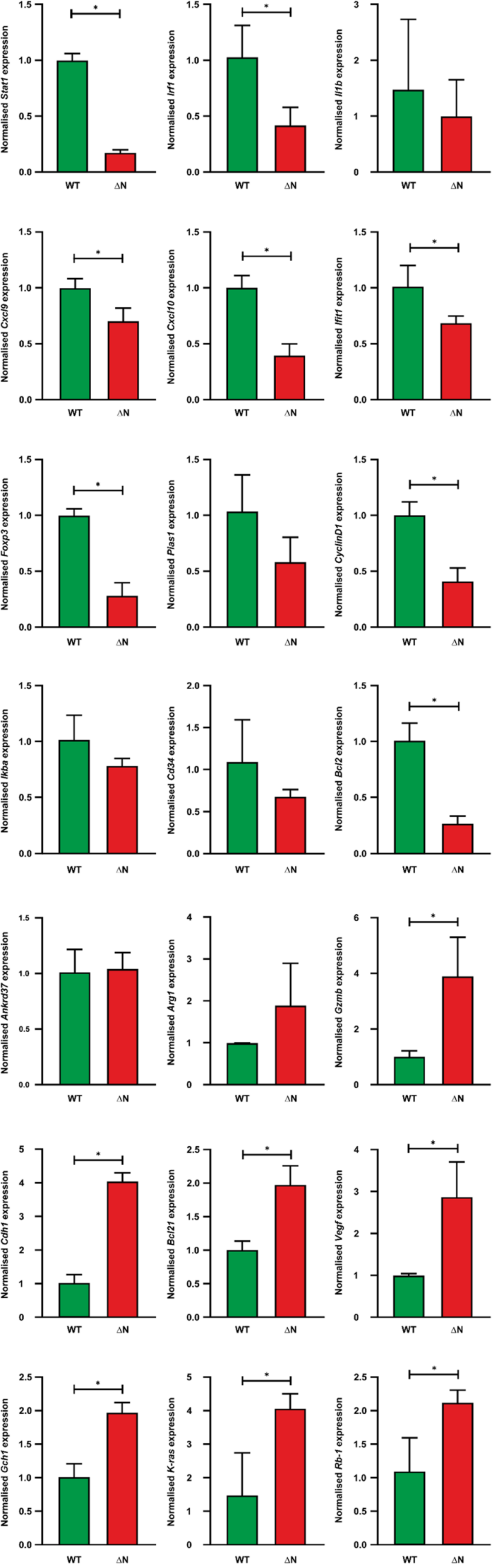
Decreased STAT1-regulated target gene expression in NHL tumor tissue. Quantitative real-time PCR was used to measure mRNA expression from spleen tissue of WT-expressing animals versus lymphoma tissue of STAT1-∆N-expressing animals. The tissues were homogenized using beads before RNA extraction and subsequent cDNA synthesis. All genes were normalized to the expression of the housekeeping gene *Gapdh*. The experiments were performed in triplicate

To confirm the transcriptional differences between NHL tumors and normal spleens, tumor samples obtained from spleens of STAT1-∆N-expressing mice versus healthy controls expressing the WT protein were examined in an independent experiment. The tissues were homogenized using beads before RNA extraction and subsequent cDNA synthesis. Real-time qPCR was performed for a set of STAT1 and STAT3 target genes identified from the RNA-seq dataset. All genes were normalized to *Gapdh* expression levels. The majority of the STAT1-target genes examined showed significantly reduced transcriptional activity in tumor tissue compared to normal tissue (Fig. 5). However, the relative mRNA expression levels of *Arg1*, *Cdh1*, *Gzmb*, *Bcl21*, *Vegf*, *Gch1*, *Kras*, and *Rb1*, all of which are known STAT3-target genes, were higher in STAT1-∆N-expressing tumors than in the unaffected spleens, pointing to the oncogenic effect mediated by STAT3 in the absence of functional STAT1 expression (Fig. 5).

## Discussion

The results presented in this study demonstrate that STAT1-∆N mice older than six months develop significant splenomegaly due to multifocal extranodal tumor formation, a pathology not observed in WT littermates. Our data show that the CD3- and CD20-negative tumor formations resembled characteristic features of NHL and histopathologically exhibited immunopositivity for both tyrosine-phosphorylated STAT1 and STAT3. Moreover, in isolated tumor cells, the N-terminally truncated STAT1 and, to a much lesser extent STAT3, were hyperphosphorylated irrespective of cytokine stimulation, as compared to non-transformed spleen cells from their WT-expressing littermates. The elevated tyrosine phosphorylation levels did not further increase following IFNγ and/or LPS stimulation of isolated tumor cells. Even before cytokine stimulation, extracts from tumor cells demonstrated enhanced DNA-binding activity of STAT1-∆N to a high-affinity GAS site, confirming a basal activation level. Despite elevated STAT1 tyrosine phosphorylation, real-time PCR analysis revealed reduced expression of STAT1-regulated genes and conversely elevated induction of STAT3-mediated genes in tumor samples compared to non-transformed spleen cells from WT animals due to the disrupted nuclear import of the mutant STAT1. The N-terminus of STAT1 contributes to its nuclear import, although the underlying mechanisms have not been resolved yet, as the nuclear localization signal within the STAT1 protein resides in the DNA-binding domain [31].

In comparison to STAT1-WT-expressing cells from normal spleens, isolated tumor cells stimulated with either IFNγ, LPS, or the combination of both showed a shift towards upregulation of STAT3-activated genes, such as *Arg1*, *Ccl2*, and *Cdh1*. In contrast to the WT protein, mutant STAT1-∆N failed to accumulate in the nucleus upon cytokine stimulation. Impaired translocation of phospho-STAT1-∆N into the nucleus prevented the mutant from executing a full-fledged transcriptional response on STAT1-dependent target genes. In addition to the downregulation of STAT1 signal transduction, tumor cells showed reduced NF-κB (p65) expression and a slight decrease in the LPS-induced degradation of IκBα. In immunocompromised mice expressing the loss-of-function mutation STAT1-∆N, ongoing inflammation in the gastrointestinal tract may not only lead to hemorrhagic necrotizing typhlitis, but may also trigger splenic NHL formation. It is reasonable that uncontrolled, chronic infections in the gastrointestinal tract or other organs promoted the development of neoplasms of lymphoid tissues originating from B or T cell precursors [32, 37].

Previous studies have demonstrated that STAT1 and STAT3 can exert opposing effects on tumor development [1, 29]. Our data confirm that the balance between STAT1 and STAT3 is critical for regulating immune responses and tumorigenesis. As an interferon-driven transcription factor, STAT1 is known for its anti-microbial functions and typically acts as a tumor suppressor by inducing apoptosis and inhibiting cell proliferation at both transcriptional and non-transcriptional levels [23]. STAT1 activation leads to the upregulation of various target genes such as caspases. In contrast, the homologous STAT3 protein has been implicated in promoting oncogenesis and survival of transformed cells by activating genes such as *BclxL*, *cMyc*, and *cyclin D1*, which are involved in promoting cell survival and proliferation. [5, 41].

Our observation that the expression of the N-terminally truncated STAT1 variant is inevitably linked to increased STAT3 tyrosine phosphorylation, altered NF-κB signaling, and the development of uniform extranodal CD3- and CD20-negative NHLs underscores the concept that STAT1 and STAT3 have antagonistic effects on oncogenic pathways [1, 40]. The inability of phosphorylated STAT1-∆N to effectively enter the nucleus and activate target genes may exacerbate STAT3's oncogenic potential, further supporting the complementary nature of these transcription factors. Studies have detected STAT1-∆N expression in the livers of these STAT1-targeted mice [7, 30]. Additionally, Chan et al. demonstrated that female mice lacking functional STAT1 are prone to the spontaneously development of mammary adenocarcinomas. These mammary tumors rely on estrogen and estrogen receptors for engraftment and progression of the disease [7].

In a transgenic mouse line with a targeted mutation in the DNA-binding domain, which is different from the STAT1-∆N used here, the complete lack of STAT1 expression leads to a dysfunction of cytotoxic T lymphocytes and natural killer cells (NK), which are both essential for the elimination of malignant blood cells [2, 34]. In a transplantation model, the STAT1-deficient mice failed to reject immunogenic tumors, probably due to inhibition of antitumor cytolytic activity [10]. In the complete absence of STAT1 expression, myeloid hyperplasia was associated with the development of a malignant clone, which typically resulted in a leukemic phenotype associated with reduced lifespan [34].

In humans, the absence of T-cell (CD3) and B-cell (CD20) markers in rare and complex forms of B-cell lymphomas, known as CD3- and CD20-negative NHLs, impedes diagnosis and treatment of this subtype, which accounts for 1–2% of all B-cell lymphomas. This tumor entity is characterized by its aggressive nature, involvement of extranodal sites, resistance to chemotherapy, and unfavorable prognosis [19]. A study by Maeshima et al. confirmed that extramedullary leukemias and lymphomas lacking CD3 and CD20 markers are uncommon and challenging to diagnose [28]. In a large sample of 4977 patients diagnosed with extramedullary NHLs, the authors identified a total of 118 cases lacking detectable immunopositivity for the markers CD3 and CD20, resulting in an incidence of 2.4% for CD3^-/-^ CD20^-/- ^NHLs. The most prevalent type of extraosseous NHL was anaplastic large cell lymphoma, which accounted for 41% of the cases, followed by large B-cell/plasmablastic lymphomas (30%). Although these B-cell lymphomas did not express CD20, they stained positively for alternative markers such as MUM1, CD79a, and PAX5 [28]. The absence of CD20 can also occur either temporarily or permanently as a phenotypic shift following rituximab therapy [26, 27]. In these cases, alternative markers, such as PAX5, may be useful for diagnosis [16].

In our study, we observed a significant decrease in NF-κB (p65) protein levels in tumor cells expressing the STAT1-∆N mutation compared with their WT counterparts. This finding is crucial given the vital function of NF-κB in modulating immune responses. Previous studies have emphasized the multifaceted role of NF-κB in cancer biology [9, 11, 14, 15]. Although NF-κB contributes to the defense of the immune system against transformed cells, its persistent activation in various cancers can facilitate tumor development. Specifically, prolonged inflammatory conditions in tumors are often linked to increased NF-κB activity and crosstalk with STAT3 signaling, thereby supporting cancer cell survival and proliferation [11, 14, 15]. NF-κB activation alters inflammatory stimuli into proliferative signals and induces tumor formation. Moreover, altered NF-κB levels may indicate a shift from an initial inflammatory response to a microenvironment in which tumor cells evade immune detection. This is in line with previous findings that tumors often manipulate NF-κB signaling to circumvent immune responses [38].

In summary, the data confirm that STAT1 and STAT3 have opposing roles in the cancer biology of NHLs. A dysfunctional STAT1 variant, which is unable to translocate into the nucleus upon cytokine stimulation and fails to inhibit STAT3, can trigger lymphomagenesis, as STAT3 becomes more active and contributes to lymphoid malignancy and tumor progression. This antagonistic interaction between STAT1 and STAT3 is crucial for understanding their complex roles in the biology of neoplasms and highlights potential therapeutic targets for modulating these pathways.

## Supplementary Information

Supplementary Material 1. Supplemental Figure 1. A Top terms of differentially regulated Kyoto Encyclopedia of Genes and Genomes pathways in comparison between STAT1-∆N-expressing NHL tumor cells and normal spleen cells from WT mice. B Overview of the “cytokine-cytokine receptor interaction” scheme differentially regulated in NHL samples versus normal spleen tissue.

Supplementary Material 2.

Supplementary Material 3.

## Data Availability

No datasets were generated or analysed during the current study.
