# Supplementary figures and images for: N-terminal truncation of STAT1 transcription factor causes CD3- and CD20-negative non-Hodgkin lymphoma through upregulation of STAT3-mediated oncogenic functions
Source: Cell Commun Signal. 2025 Apr 26;23:201. doi: 10.1186/s12964-025-02183-2 (PMC12034123; doi:10.1186/s12964-025-02183-2)

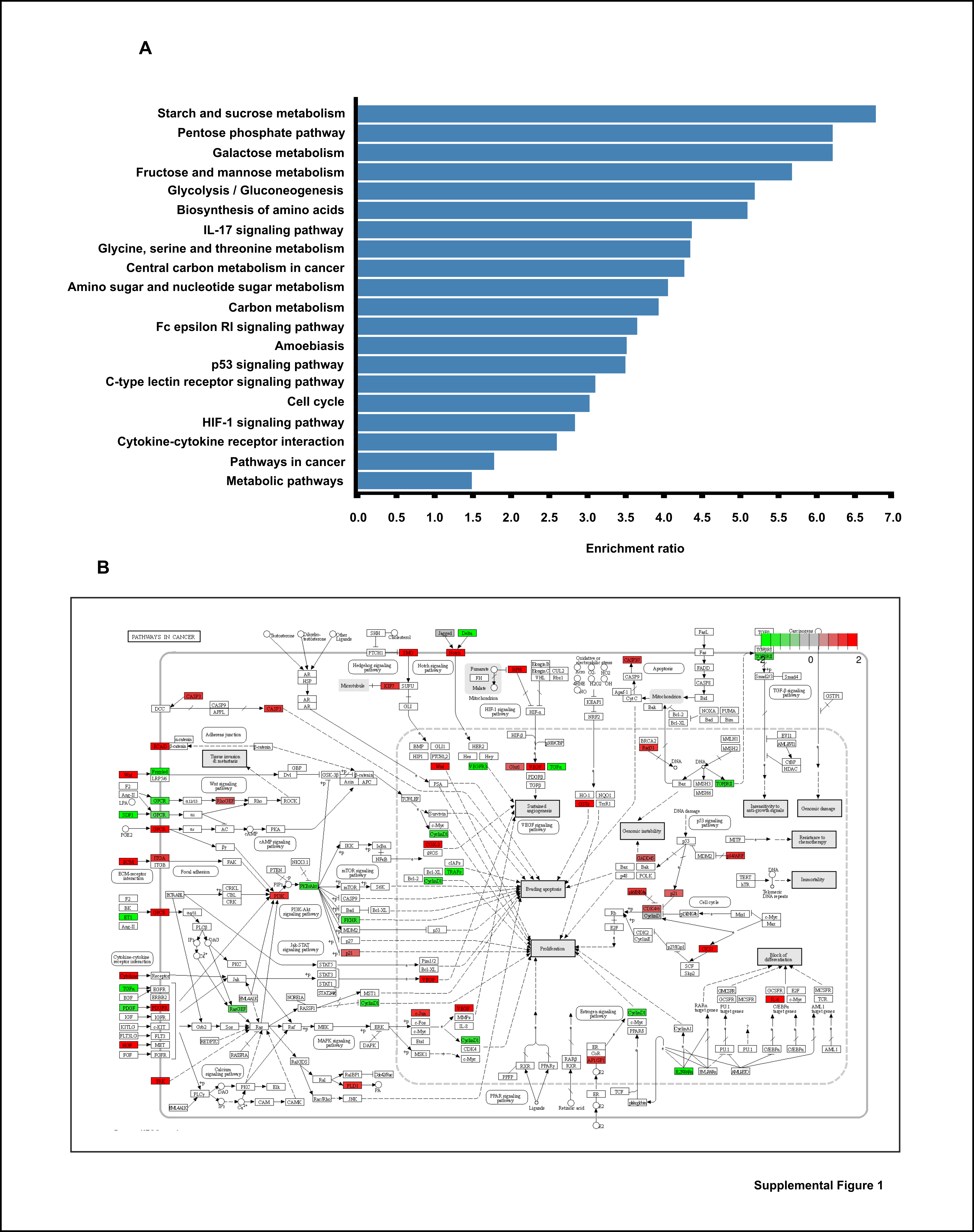

Supplement: Supplementary file 1 — Supplementary Material 1. Supplemental Figure 1. A Top terms of differentially regulated Kyoto Encyclopedia of Genes and Genomes pathways in comparison between STAT1-∆N-expressing NHL tumor cells and normal spleen cells from WT mice. B Overview of the “cytokine-cytokine receptor interaction” scheme differentially regulated in NHL samples versus normal spleen tissue. [file 12964_2025_2183_MOESM1_ESM.tif]
