# Supplementary material for: N-terminal truncation of STAT1 transcription factor causes CD3- and CD20-negative non-Hodgkin lymphoma through upregulation of STAT3-mediated oncogenic functions
Source: Cell Commun Signal. 2025 Apr 26;23:201. doi: 10.1186/s12964-025-02183-2 (PMC12034123; doi:10.1186/s12964-025-02183-2)
Supplement: Supplementary file 2 — Supplementary Material 2. [file 12964_2025_2183_MOESM2_ESM.docx]

**Supplemental Table 1:**

VEGFF; 5‘-CAGGCTGCTCTAACGATGAA-‘3

VEGFR; 5‘-CAGGAATCCCAGAAACAACC-‘3

IL-6F; 5‘-TCCAGTTGCCTTCTTGGGAC-‘3

IL-6R; 5‘-GTACTCCAGAAGACCAGAGG-‘3

TNF-aF; 5‘-CATCTTCTCAAAATTCGAGTGACAA-‘3

TNF-aR; 5‘-TGGGAGTAGACAAGGTACAACCC-‘3

FOXP3F; 5‘-CCCAGGAAAGACAGCAACCTT-‘3

FOXP3R; 5‘-TTCTCACAACCAGGCCACTTG-‘3

CCL2F; 5‘-GATGCAGTTAACGCCCCACT-‘3

CCL2R; 5‘-ACCCATTCCTTCTTGGGGTC-‘3

CXCL10F; 5‘-CCACGTGTTGAGATCATTGCC-‘3

CXCL10R; 5‘-GAGGCTCTCTGCTGTCCATC-‘3

IFIT1F; 5‘-AGCAACCATGGGAGAGAATGC-‘3

IFIT1R; 5‘-CCTTTCAGGTGCCTCACGTA-‘3

IFIT3F; 5‘-CTGAACTGCTCAGCCCACA-‘3

IFIT3R; 5‘-TTCCCGGTTGACCTCACTCA-‘3

BCL2L1F; 5‘-GCCTTTTTCTCCTTTGGCGG-‘3

BCL2L1R; 5‘-TCCACAAAAGTGTCCCAGCC-‘3

STAT3F; 5‘-AATTATGCATGGAGGCGTGT-‘3

STAT3R; 5‘-GTACCTCAGCGATCCGGTTA-‘3

INOSF; 5‘-GAACCTACCAGCTCACTCTG-‘3

INOSR; 5‘-GATGTGCTGAAACATTTCCT-‘3

GAPDHF; 5‘-AATGGTGAAGGTCGGTGTGAAC-‘3

GAPDHR; 5‘-GAAGATGGTGATGGGCTTCC-‘3

IRF1F; 5‘-ACCCTGGCTAGAGATGCAGA-‘3

IRF1R; 5‘-TGCTTTGTATCGGCCTGTGT-‘3

CXCL9F; 5‘-GGAGTTCGAGGAACCCTAGT-‘3

CXCL9R; 5‘-AGGCAGGTTTGATCTCCGTT-‘3

CyclinD1F; 5‘-TCAAGTGTGACCCGGACTG-‘3

CyclinD1R; 5‘-ATGTCCACATCTCGCACGTC-‘3

BCL2F; 5‘-GAACTGGGGGAGGATTGTGG-‘3

BCL2R; 5‘-GCATGCTGGGGCCATATAGT-‘3

PIASF; 5‘-TGCCTTGACACCACAAC-‘3

PIASR; 5‘-GCTTTGGTTCCACACCG-‘3

IL-1BF; 5‘-GAAATGCCACCTTTTGACAGTG-‘3

IL-BR; 5‘-TGGATGCTCTCATCAGGACAG-‘3

IKBF; 5‘-GAAGCCGCTGACCATGGAA-‘3

IKBR; 5‘-GATCACAGCCAAGTGGAGTGGA-‘3

K-rasF; 5‘-CAAGAGCGCCTTGACGATACA-‘3

K-rasR; 5‘-CCAAGAGACAGGTTTCTCCATC-‘3

CD34F; 5‘-AGGCTGATGCTGGTGCTAG-‘3

CD34R; 5‘-AGTCTTTCGGGAATAGCTCTG-‘3

Rb1F; 5‘-ACTCCGTTTTCATGCAGAGAACTAA-‘3

Rb1R; 5‘-GAGGAATGTGAGGTATTGGTGACA-‘3

GCH1F; 5‘-AGGAAGGGTCCATATTGGCT-‘3

GCH1R; 5‘-CCACCGCAATCTGTTTGGTG-‘3

STAT1F; 5‘-TGGTGAAATTGCAAGAGCTG-‘3

STAT1R; 5‘-CAGACTTCCGTTGGTGGATT-‘3
