# Supplementary material for: N-terminal truncation of STAT1 transcription factor causes CD3- and CD20-negative non-Hodgkin lymphoma through upregulation of STAT3-mediated oncogenic functions
Source: Cell Commun Signal. 2025 Apr 26;23:201. doi: 10.1186/s12964-025-02183-2 (PMC12034123; doi:10.1186/s12964-025-02183-2)
Supplement: Supplementary file 3 — Supplementary Material 3. [file 12964_2025_2183_MOESM3_ESM.pdf]

pSTAT1 and STAT1 Blot 1

WT STAT1-ΔN

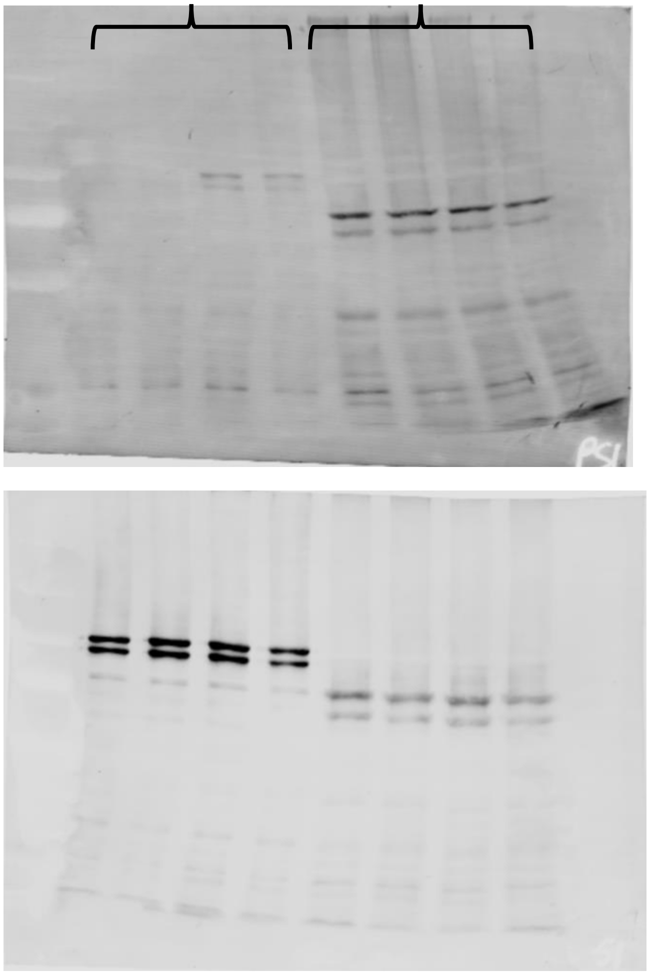

pSTAT1

STAT1

pSTAT1 and STAT1 Blot 2

WT STAT1-ΔN

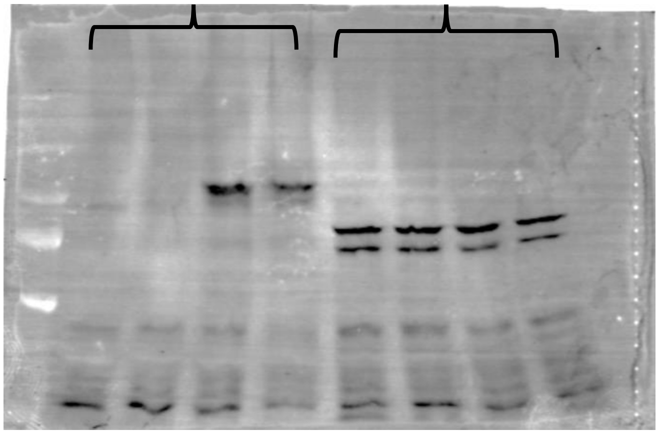

pSTAT1

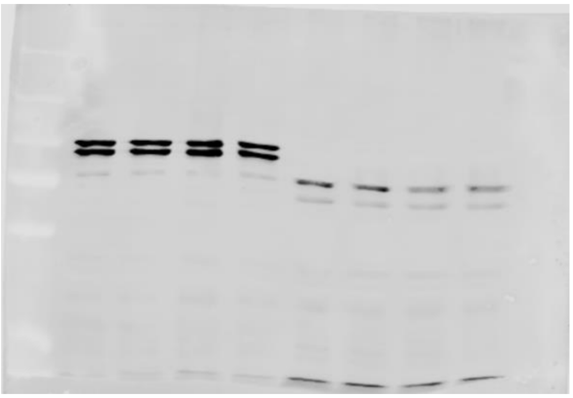

STAT1

pSTAT1 and STAT1 Blot 3

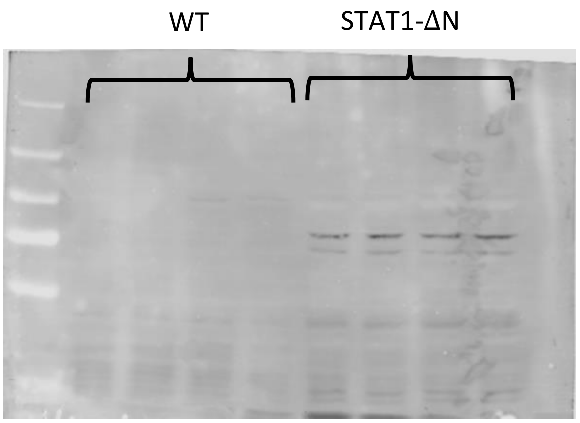

pSTAT1

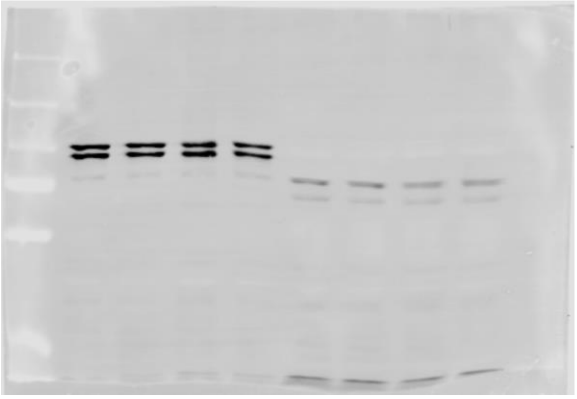

STAT1

pSTAT3 and STAT3 Blot 1

WT STAT1-ΔN

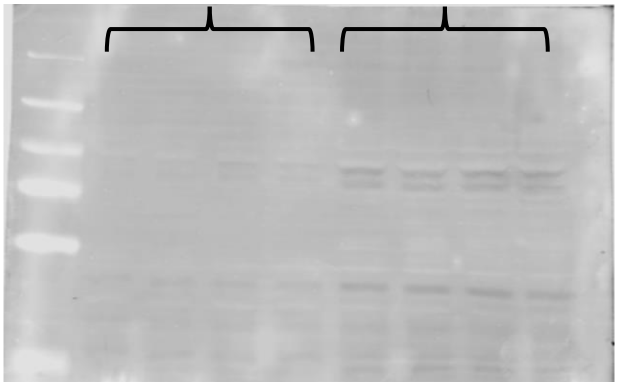

pSTAT3

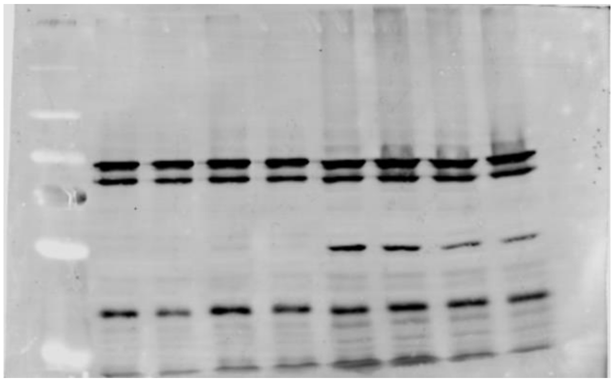

STAT3

pSTAT3 and STAT3 Blot 2

WT STAT1-ΔN

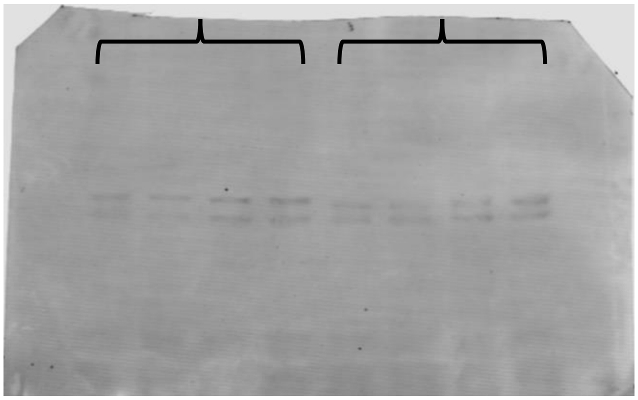

pSTAT3

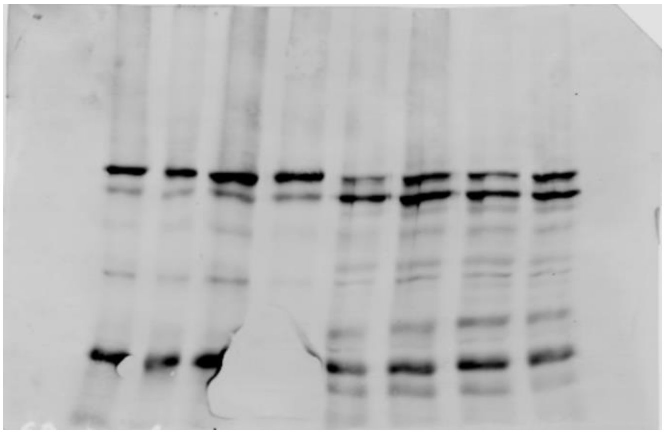

STAT3

pSTAT3 and STAT3 Blot 3

WT STAT1-ΔN

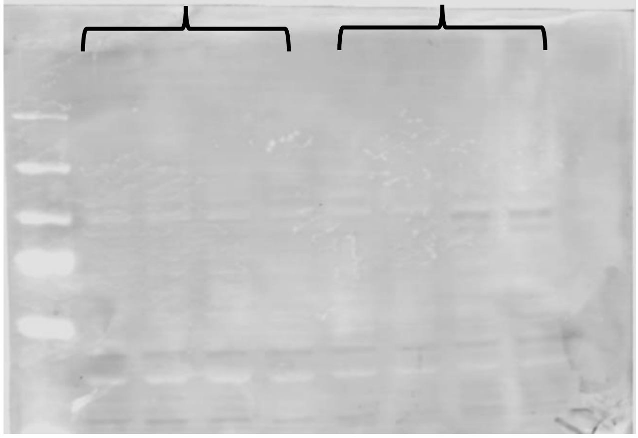

pSTAT3

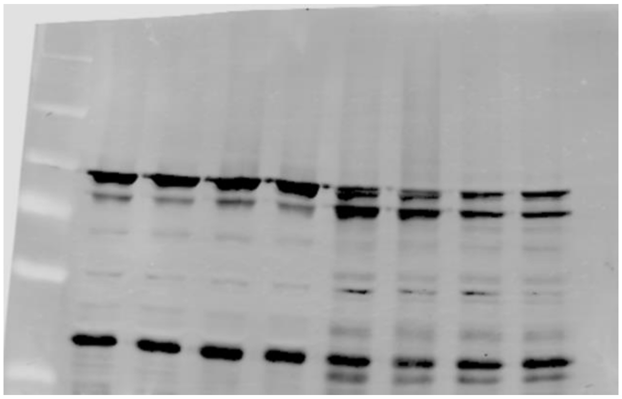

STAT3

IκBα Blot 1

WT STAT1-ΔN

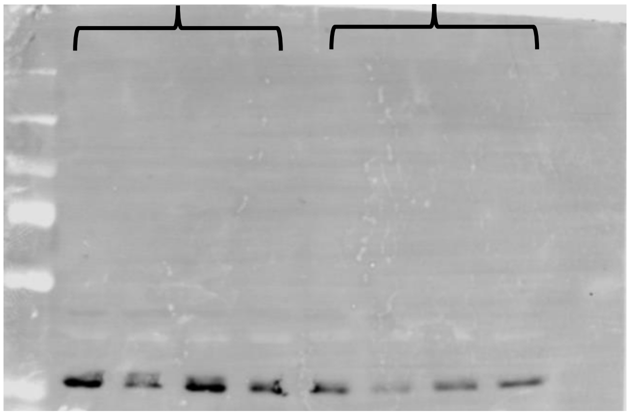

IκBα

IκBα Blot 2

WT STAT1-ΔN

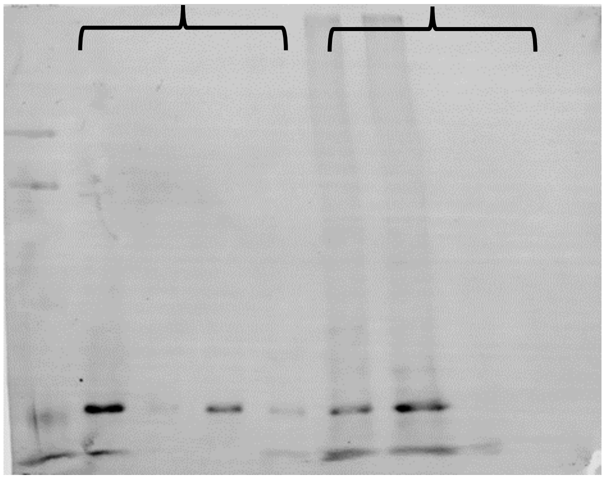

IκBα

IκBα Blot 3

WT STAT1-ΔN

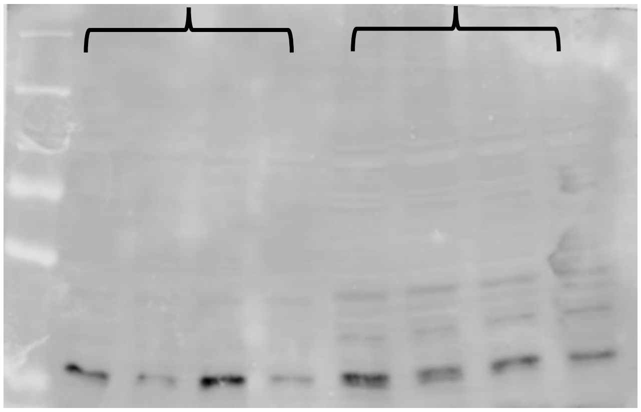

IκBα

pP65 Blot 1

WT STAT1-ΔN

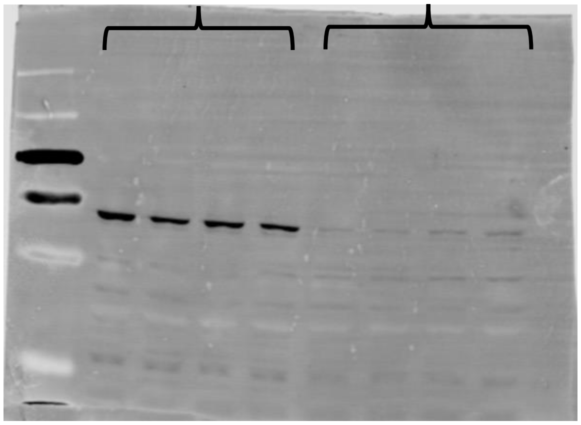

p65

p65 Blot 2

WT STAT1-ΔN

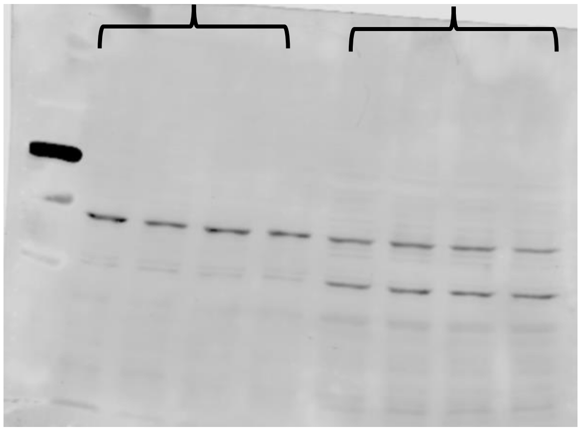

p65

p65 and GAPDH Blot 3

WT STAT1-ΔN

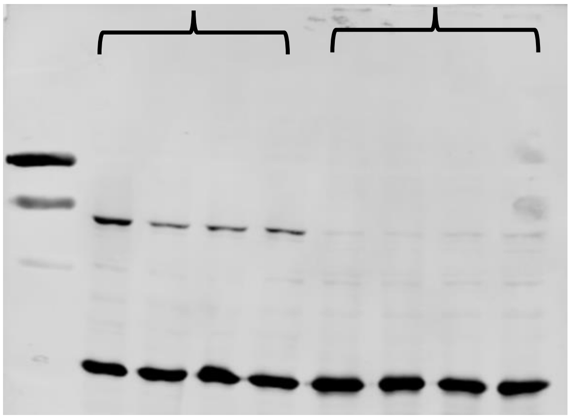

p65 and GAPDH

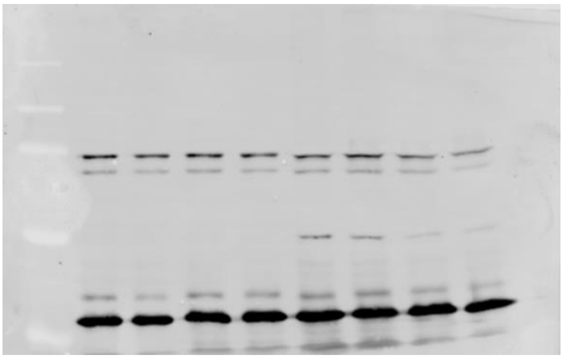

GAPDH

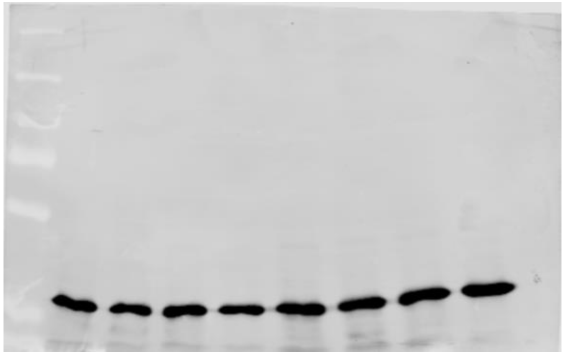

GAPDH

WT STAT1-ΔN

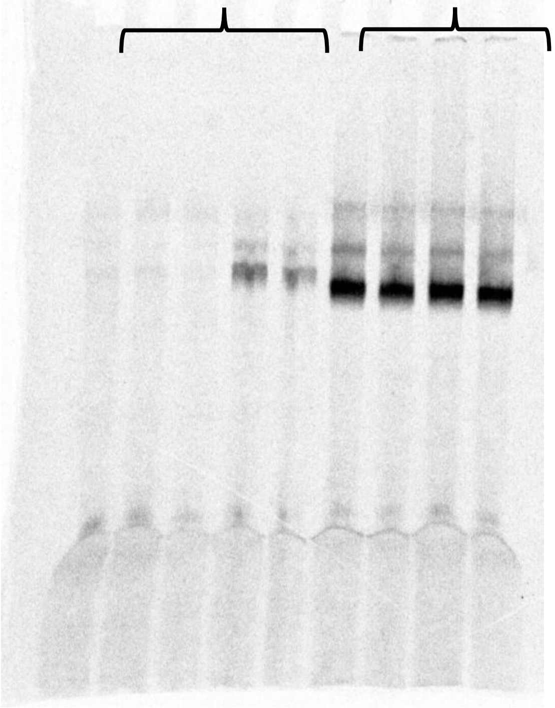

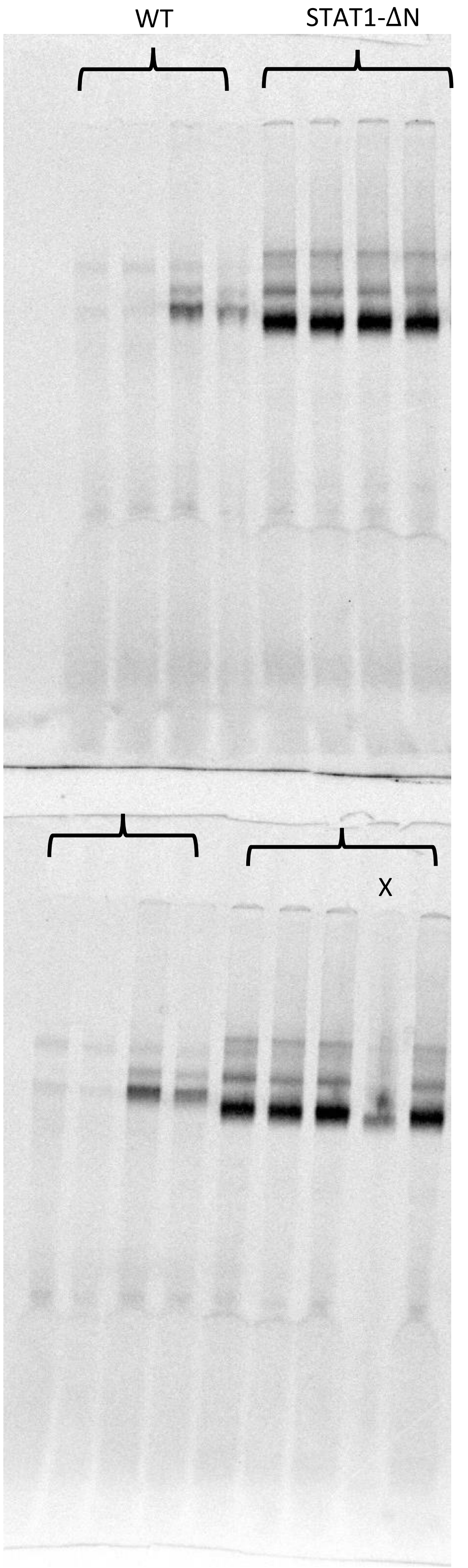

X: lane misloaded
